# The Educator Within: A Systematic Review of Professional Identity Formation in Health Professions Education

**DOI:** 10.12669/pjms.42.1.13674

**Published:** 2026-01

**Authors:** Shams Nadeem Alam, Syeda Kauser Ali, Lubna Ansari Baig

**Affiliations:** 1 Shams Nadeem Alam Associate Professor, Department of Medical Education, Baqai Medical University, Karachi, Pakistan; 2 Syeda Kauser Ali Chairperson, Institute of Medical Education, Jinnah Sindh Medical University, Karachi, Pakistan; 3 Lubna Ansari Baig, PhD Program Director, University of Lahore, Lahore, Pakistan

**Keywords:** Educator identity, Faculty development, Health professions education, Professional identity formation, Qualitative systematic review, Thematic synthesis

## Abstract

**Objective:**

To explore the individual and community-level factors influencing the professional identity formation (PIF) of health professions educators and educationists and examine how these factors influence educator identity within professional communities.

**Methodology:**

This qualitative systematic review followed the SPIDER and PICO frameworks to identify relevant empirical literature published between 2005 to 2024. Four databases (PubMed, ERIC, CINAHL, and PsycINFO) were searched. Studies were included if they employed qualitative or mixed methods with significant qualitative components and focused on health professions educators. Data were synthesized using thematic synthesis following Thomas and Harden’s three-stage approach. Quality appraisal was conducted using the CASP checklist, and inclusion required a minimum score of 12 out of 18 across six evaluative criteria.

**Results:**

Sixteen studies were included, representing diverse geographic and disciplinary contexts. Thematic synthesis revealed four core domains influencing PIF: (1) transitions into educator roles, (2) individual-level drivers such as motivation and reflective agency, (3) community and institutional enablers including mentorship and recognition, and (4) psychological processes such as impostor phenomenon and identity affirmation. These factors interact dynamically within socio-cultural contexts to shape educator identity.

**Conclusion:**

Professional identity formation in health professions educators is a multidimensional, non-linear process shaped by personal motivation, institutional culture, and emotional validation. Faculty development programs must explicitly address identity support alongside pedagogical training to foster sustainable educator roles and academic engagement. Institutional policies should recognize and reward educational contributions to strengthen teaching legitimacy in clinical academic settings.

## INTRODUCTION

The formation of a professional identity (PI) among health professional educators and educationists represents a significant yet underexplored dimension of health professions education (HPE). While substantial literature exists concerning identity development among clinicians, limited systematic attention has been paid to the educators who support and lead such training. These individuals often enter educational roles through varied trajectories, bringing with them existing professional identities, typically rooted in clinical practice, research, or academic administration. As they transition into roles centered around teaching and faculty development, these pre-existing identities interact with new expectations and values associated with the educator role.1,2

Professional identity has been described as a dynamic, evolving self-concept that emerges through sustained engagement within a particular professional community.3 Within health professions education, such identity development is complex, influenced not only by individual-level factors, such as motivation, self-perception, and prior experiences, but also by community factors, including workplace culture, peer interactions, institutional norms, and the availability of mentoring or professional development opportunities.4,5 This dual influence reflects a broader understanding of identity as socially constructed, situated within and shaped by participation in communities of practice.

Emerging empirical work suggests that for many educators, professional identity is negotiated through tensions between the existing identities (clinical identity in most cases) and the developing sense of self as an educationist. Experiences of identity dissonance, ambiguity, and marginalization are not uncommon, particularly in academic environments where teaching contributions are less visibly valued compared to research outputs.6,7 Conversely, identity consolidation appears more likely where educators can engage meaningfully with others who value educational work, and where structures exist to support recognition, collaboration, and growth in pedagogical competence.8,9

The socio-cultural lens provides an important foundation for understanding these dynamics. From this perspective, identity is not seen as a fixed characteristic but as fluid and formed through engagement with cultural tools, practices, and communities over time.10 In this context, the role of community, peers, institutional leadership, learners, and disciplinary networks, is pivotal in affirming identity, and enabling transformation from one professional stance to another. Furthermore, individual-level factors, including agency, reflective capacity, and resilience, interact with these communal influences to shape how educators interpret their role, locate their professional self, and develop a sustained commitment to educational work.11

Despite increasing recognition of the importance of these processes, there remains an absence of comprehensive synthesis examining how individual and community factors coalesce to influence identity development in this specific professional group. Existing evidence, though growing, remains fragmented and predominantly qualitative in nature, necessitating a systematic approach to map and integrate findings. Moreover, little is known about how these identity-shaping factors interact over time to form coherent narratives of professional belonging and legitimacy among health professions educators.

Although prior reviews12,13 describe aspects of educator identity, they do not synthesise how individual, institutional, and psychological processes interact specifically among health professions educators and educationists across diverse contexts. The current review addresses this gap by integrating these dimensions and presenting an updated conceptual model.

In response to this gap, the present systematic review aims to explore the key individual and community-level factors that influence the professional identity formation of health professional educators and educationists. A further objective is to examine how these factors interact to shape their identities as legitimate members of professional communities. By synthesizing empirical studies across diverse HPE contexts, this review seeks to contribute a nuanced understanding that may inform the design of supportive faculty development initiatives, mentorship models, and institutional frameworks that nurture educator identity within the health professions.

## METHODOLOGY

This qualitative systematic review was conducted to examine the factors influencing the professional identity formation (PIF) of health professional educators and educationists. Two structured frameworks guided the review design: an adapted **P**opulation, **I**ntervention, **C**omparison, and **O**utcome (PICO) structure for qualitative evidence and the SPIDER tool (**S**ample, **P**henomenon of **I**nterest, **D**esign, **E**valuation, **R**esearch type), designed by Cooke et al.14, specifically to identify relevant qualitative and mixed-methods studies. These frameworks provided a robust conceptual foundation for framing the research questions and informing the search strategy.15 These frameworks provided a robust conceptual foundation for framing the research questions and informing the search strategy.16 *The review addressed two central questions:*

What are the key individual and community factors that influence the professional identity formation of health professional educators and educationists?

How do these factors interact to shape their identities as members of professional communities?

### Eligibility criteria: Following the SPIDER tool15, the eligibility criteria were defined as follows:

- Sample: Health professional educators and educationists, including those from fields such as medicine, nursing, pharmacy, and allied health.
- Phenomenon of Interest: Professional identity formation, specifically the influence of individual and community-level factors
- Design: Qualitative or mixed-method studies with significant qualitative components (e.g., interviews, focus groups, ethnographies).
- Evaluation: Studies that explored perceptions, experiences, or reflections related to identity formation.
- Research Type: Peer-reviewed empirical research published in English between 2005 and 2024.
- Studies were excluded if they focused solely on student or clinical identities, lacked qualitative data, or did not provide relevant insights into the educator identity formation process.17

### Search strategy: A comprehensive search was conducted across four databases: PubMed, ERIC, CINAHL, and PsycINFO. Search terms were constructed using SPIDER and adapted PICO elements, including:

- Population: “health professional educators,” “medical educators,” “health professions educationists.
- Interest: “professional identity,” “identity formation,” “individual factors,” “community factors.
- Context: Professional experiences and settings without direct comparisons.
- Outcome: “professional development,” “educational identity,” “sense of belonging.”
- Boolean operators and truncations were used to enhance sensitivity. Reference lists of key articles were also reviewed for additional relevant studies.

### Study selection and Review process:

The selection process followed the Preferred Reporting Items for Systematic reviews and Meta-Analyses extension for Scoping Reviews (PRISMA-ScR) guidelines.18,19 Three reviewers independently screened titles and abstracts, followed by full-text review against eligibility criteria. Discrepancies were resolved through consensus. A PRISMA flow diagram was used to document the search and selection process.

### Quality appraisal and Scoring criteria:

All included studies were appraised using the **Critical Appraisal Skills Programme (**CASP) Qualitative Checklist.20 To structure inclusion decisions, a scoring framework was applied across six criteria:

- Relevance to research questions.
- Methodological quality.
- Study design and data collection methods.
- Depth of analysis.
- Contribution to understanding professional identity formation.
- Relevance to professional community engagement.

Each criterion was scored on a three points scale (1 = Does not meet, 2 = Partially meets, 3 = Fully meets), resulting in a maximum possible score of 18.21 The cutoff score for inclusion was set at 12, with a minimum score of 2 required on both relevance to research questions and methodological quality. Borderline cases (scores of 11-12) underwent secondary screening for thematic alignment and insight value, enabling flexibility without compromising rigor.

### Data Extraction:

A structured data extraction form was developed to capture the following data.22

- Author(s), year, country.
- Study design and data collection methods.
- Participant characteristics.
- Themes related to individual and community factors, mentoring, support, and engagement within the professional community.
- Theoretical framework.
- Key findings relevant to individual and community identity-shaping factors.

### Thematic Synthesis:

The findings were synthesized using thematic synthesis as outlined by Thomas and Harden allowing for the integration of qualitative evidence across diverse contexts.21 The synthesis unfolded in three iterative stages:

- Initial coding: Relevant findings from each included study were coded line-by-line to identify core meanings related to professional identity formation.
- Descriptive theme development: Codes were grouped into broader descriptive categories capturing recurrent patterns related to individual and community-level influences.
- Analytical theme refinement and validation: Three researchers independently reviewed the descriptive themes and final synthesis, offering critical reflections on coherence, thematic structure, and interpretive depth.

Their involvement ensured credibility, enhanced reflexivity, and helped refine conceptual clarity. This collaborative review process supported transparency and minimized individual bias in interpretation. This staged approach enabled the construction of higher-order insights into how identity is shaped, negotiated, and sustained by health professional educators across various settings.22,23

### Reporting Standards:

The review adhered to PRISMA-ScR reporting standards24 ensuring transparency in the identification, selection, appraisal, and synthesis of studies.

## RESULTS

### Description of Included Studies:

Sixteen articles were included in the final synthesis. These studies encompassed a variety of research designs, including qualitative research (e.g., interviews, focus groups, narrative analysis), scoping reviews, and systematic reviews. They also represented diverse geographic contexts including the Netherlands, United States, Indonesia, Australia, the United Kingdom, Singapore, and Iran. Study participants ranged from early-career educators and postgraduate students to experienced clinician-educators transitioning into formal teaching roles.

The studies explored a wide array of educational settings and identity journeys. Some investigated formal development programs25,26 while others focused on informal mentoring and community-based support systems.12,27 A summary of these studies, including their methodological characteristics and key contributions, is provided in Table-I.

**Table-I:** Summary of Included Studies on Professional Identity Formation of Health Professions Educators.

No.	Title / Author(s) & Year	Study Design & Participants	Focus / Context	Key Findings / Contribution
1	The Health Professions Education Pathway: Preparing Students, Residents, and Fellows to Become Future Educators — *Chen HC et al., 2017*	Qualitative program evaluation of students, residents, and fellows	Longitudinal formal educator-preparation program	Structured faculty development enhances educator legitimacy and fosters early educator identity.
2	Translating Faculty Development into Practice and Professional Identity: The Lived Experience of Clinical Educators — *Bartle E et al., 2025*	Qualitative phenomenological study with clinical educators	Longitudinal faculty development and identity transformation	Reflective, practice-linked programs consolidate educator identity through institutional recognition and community belonging.
3	Developing a Teacher Identity in the University Context: A Systematic Review of the Literature — *van Lankveld T et al., 2016*	Systematic review of academic teacher-identity studies	University-based teacher identity formation	Identified community, feedback, and institutional culture as core enablers of educator identity.
4	Novice Clinician-Educator Professional Identity Formation Through a Longitudinal Mentorship: A Qualitative Study — *Fong W & Jones L., 2024*	Longitudinal qualitative study of novice clinician-educators	Mentorship-based identity development	Sustained mentorship enables transformation from clinician to educator and mitigates identity dissonance.
5	Exploring the Tensions of Being and Becoming a Medical Educator — *Sethi A et al., 2017*	Qualitative exploration with medical educators	Transition challenges in medical education roles	Highlighted competing clinical-educator identities and need for reflective support.
6	From Clinician to Educator: A Scoping Review of Professional Identity and the Influence of Impostor Phenomenon — *Freeman KJ et al., 2022*	Scoping review	Professional identity and impostor feelings	Impostor phenomenon identified as key barrier to identity consolidation and wellbeing.
7	The Journey from Clinician to Undergraduate Medical Educator Involves Four Patterns of Transformation — *Riveros-Perez E & Rodriguez-Diaz J., 2017*	Narrative inquiry with clinician-educators	Transition to undergraduate teaching	Outlined four transformation patterns emphasizing reflective learning and mentorship.
8	Making the Leap to Medical Education: A Qualitative Study of Medical Educators’ Experiences — *Browne J et al., 2018*	Qualitative interviews with new medical educators	Experience of entering medical education	Meaning-making and institutional alignment underpin sustained educator identity.
9	Becoming a Medical Educator: Motivation, Socialisation and Navigation — *Bartle E & Thistlethwaite J., 2014*	Qualitative interviews with medical educators	Motivation and socialisation into teaching roles	Altruism and desire to contribute to future generations drive intrinsic motivation.
10	Developing Professional Identity in Nursing Academics: The Role of Communities of Practice — *Andrew N et al., 2009*	Case study of nursing faculty	Role of communities of practice in identity formation	Belonging and collegiality strengthen academic identity and teaching confidence.
11	Integrating the Teaching Role into One’s Identity: A Qualitative Study of Beginning Undergraduate Medical Teachers — *van Lankveld T et al., 2017*	Qualitative study with novice medical teachers	Early-career teacher identity development	Reflection and peer feedback facilitate internalization of educator role.
12	Fitting In While Standing Out: Professional Identity Formation, Imposter Syndrome, and Burnout in Early-Career Faculty Physicians — *Vaa Stelling BE et al., 2023*	Qualitative study of early-career physicians	Relationship of impostor syndrome to PIF	Demonstrated tension between belonging and distinctiveness; impostorism linked to burnout risk.
13	Influences on and Characteristics of the Professional Identity Formation of Clinician-Educators: A Qualitative Analysis — *Triemstra JD et al., 2021*	Qualitative thematic analysis	Determinants of clinician-educator identity	Recognition, validation, and institutional culture critical for educator legitimacy.
14	Clinical Teachers’ Professional Identity Formation: An Exploratory Study Using the 4S Transition Framework — *Sueningrum AASA et al., 2022*	Exploratory qualitative study	Transitions of clinical teachers	Reflection, support, and belonging foster positive identity transition.
15	Authoring the Identity of Learner Before Doctor in the Figured World of Medical School — *Stubbing E et al., 2018*	Qualitative interpretive study using figured-worlds lens	Learner-to-doctor identity formation	Showed relational, cultural, and dialogic processes of identity construction.
16	The Development of the Personal Self and Professional Identity in Learning to Teach — *Rodgers CR & Scott KH., 2008*	Conceptual / theoretical analysis	Foundational theory for educator identity formation	Provided conceptual grounding for reflective and relational identity development.

### Thematic findings:

Analysis of the selected studies revealed four interrelated themes that contributed to professional identity formation among health profession educators.

### Transitions to educator identity:

The transition into an educator role was consistently described as a dynamic, often non-linear journey. Many studies highlighted tensions between previous clinical or research roles and new teaching responsibilities, requiring individuals to reconcile competing professional identities.13,28 For some, this transformation followed a reflective, developmental process grounded in self-exploration and learning-by-doing.29 Others entered medical education intentionally through formal pathways25 or reported transformative experiences during teaching that led to identity shifts.26,30 This further illustrated that novice clinician-educators often perceived themselves primarily as clinicians at the outset, but through longitudinal mentorship and reflective engagement, gradually began to integrate educational responsibilities into their evolving professional identity.

### Individual-level factors:

Several individual drivers and mechanisms were identified:

### Motivation and meaning:

Personal passion, altruism, and the desire to impact future generations were key motivational factors. Educators expressed a strong sense of meaning and purpose in teaching, as presented by Bartle31 and Andrews.

### Reflection and agency:

Reflective practice served as a tool to consolidate self-perception as educators. Van Lankveld et al.33 showed that novice teachers actively engaged in sense-making to integrate teaching into their professional identities. Bartle et al.26 reinforced this in 2025 by demonstrating that longitudinal faculty development programs that incorporated structured reflection and opportunities for application enabled clinical educators to align teaching with their professional values, deepening their educator identity.

### Navigating doubt and Impostor feelings:

The impostor phenomenon emerged as a notable psychological barrier. Early-career educators often felt underqualified, despite external validation as shown by Vaa Stelling et al.34 and Freeman et al.

### Community and Institutional-level factors:

The institutional environment and peer interactions played a crucial role:

### Mentorship and Peer support:

Informal communities of practice were essential for affirmation and professional growth. These communities fostered a sense of belonging and collective identity as delineated by van Lankveld et al.12 in 2016 and Andrew et al.32 in 2009. Fong and Jones27 in 2024 emphasized the role of mentorship in this process, showing how novice clinician-educators relied on sustained guidance and collegial exchange to negotiate the tensions between clinical and educator identities.

### Recognition and Validation:

Studies emphasized that formal acknowledgment of educational contributions enhanced identity development. Lack of recognition was associated with marginalization and internal conflict as identified by Triemstra et al.35 in 2021

### Leadership and Cultural support:

Chen and colleagues25 and then Sethi et al.28 showed that supportive leadership and organizational cultures that prioritized education were strong enablers. Institutional endorsement of educator roles legitimized the identity transition. Bartle et al.26 in 2025 added further evidence by demonstrating that clinical educators who participated in longitudinal faculty development programs experienced stronger identity consolidation when embedded in institutional cultures that promoted reflection, community engagement, and recognition of educational work.

### Psychological processes and Mechanisms:

Professional identity formation was also shaped by deep-seated emotional and psychological processes:

- *Appreciation and Connectedness:* Emotional rewards, such as being appreciated by students or recognized by peers, contributed to increased self-worth and identity affirmation a process illustrated in recent work by Sueningrum et al.36 in 2022; and Andrew et al.32 earlier in 2009.
- *Commitment and Career Satisfaction:* For several educators, the transition into teaching represented a pivotal career juncture that fostered deeper alignment between professional roles and personal values, ultimately contributing to sustained commitment and long-term career satisfaction, a developmental trajectory articulated in the work of Browne et al.30 in 2018 and Triemstra et al.35 in 2021.
- Some contextual variation across career role and resource setting was noted; these are interpreted in the Discussion

### Visual model of educator identity formation:

**Fig.1:**
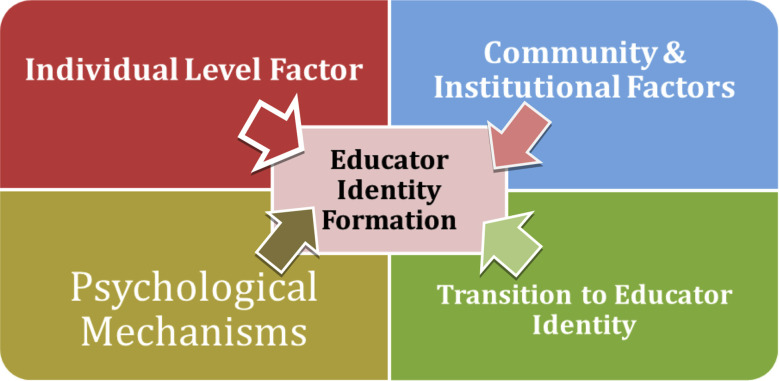
Visual model of educator identity formation illustrating the interaction between individual, institutional, and psychological factors.

Fig.1 presents a conceptual model that illustrates the interplay between individual factors, institutional support, and psychological processes in shaping educator identity. The model reflects identity formation as a non-linear, iterative process embedded in both personal reflection and social recognition. It shows how individual-level factors, community/institutional influences, and psychological mechanisms collectively contribute to the evolving identity of health professions educators. The revised model illustrates these domains as dynamically inter-connected rather than discrete, highlighting reciprocal influence among individual, community/institutional, and psychological mechanisms within broader socio-cultural settings.

## DISCUSSION

This systematic review synthesizes evidence from sixteen studies to reveal a nuanced, non-linear process of professional identity formation (PIF) in health professions educators. The findings illustrate that PIF is deeply influenced by four core domains: transitions into the educator role, individual-level drivers (such as motivation and agency), community and institutional dynamics, and psychological processes, including validation and belonging.

A particularly salient finding was the centrality of emotional and cognitive transformation during transitions into educational roles, often occurring in mid-career, post-clinical stages, where educators reevaluated their sense of purpose, legitimacy, and expertise, as demonstrated by Freeman13 in 2022 and Van Stelling et al.34 in 2023. Post-clinical refers to a phase in a health professional’s career advancement when the individual transitions away from full-time clinical practice and begins to engage more substantively in educational, academic, or administrative roles. Many educators described a shift from viewing teaching as a secondary responsibility to seeing it as a primary component of their professional identity, especially when supported by institutional structures and peer validation, as illustrated by Sueningrum et al.36 in 2022 and van Lankveld and colleagues12 in 2016. Recent work by Fong and Jones27 reinforces this path, showing that novice clinician-educators frequently perceive themselves as clinicians at the outset, which is considered as ‘Primary Identity” through longitudinal mentorship and reflective engagement gradually reconstruct their identities as educators, which can be deemed as Secondary Identity. Similarly, faculty development programs that embed reflective practice, opportunities for application, and community connections have been shown to accelerate the consolidation of an educator identity within supportive institutional cultures as shown in 2025 by Bartle et al.26 Ramani et al.37 highlight that progression from clinician-educator to educational scholar and leader depends on mentorship, reflection, and scholarly engagement, processes that mirror the mechanisms of identity consolidation identified in this review.

### Alignment and Divergence from existing literature:

The findings broadly support socio-cultural perspectives on identity as demonstrated by Cruess et al.3 in 2015, which propose that professional identity is formed through interaction, recognition, and practice. This is aligned with the idea that educators internalize identities through participation in communities of practice as shown by Wenger4 in 1998 and engagement with professional values as illustrated by Chen et al.25 in 2017. Recent evidence by Fong and Jones27 further substantiates this perspective, showing how mentorship and experiential learning enable novice clinician-educators to gradually integrate teaching into their sense of self, even when their initial identification is rooted primarily in clinical practice.

However, our synthesis reveals a growing emphasis in recent literature on the emotional labor and psychological vulnerabilities inherent in educator identity formation, areas previously underexplored. For example, impostor phenomenon emerged as a key theme, especially among early-career faculty navigating dual roles as clinicians and educators as outlined recently in 2023 by Vaa Stelling et al.34 and Freeman et al.13 in 2022. This finding diverges from earlier models of PIF which often assumed linear or rational trajectories. Instead, it highlights the emotional turbulence of transitioning identities, compounded by the hierarchical and research-dominant cultures prevalent in medical academia.

Moreover, this review highlights the tensions between recognition and invisibility. While several participants found deep meaning in teaching, lack of formal recognition (e.g., in promotion criteria or institutional narratives) often undermined their sense of legitimacy as presented by Triemstra et al.35 in 2021. Bartle et al.26 (2025) argued that contemporary scholarship demonstrates how well-designed faculty development programs can mitigate identity-related challenges by embedding structured reflection, opportunities for authentic application, and sustained community support, thereby fostering recognition and accelerating the consolidation of educator identity. This finding resonates with longstanding critiques of academic reward structures that systematically privilege research output over teaching contributions, as publicized by Hafferty & Castellani in 2011, thereby reinforcing the marginalization of educator identities and underscoring the imperative to reconceptualize faculty development through frameworks identified by researchers38 that explicitly foregrounds recognition and legitimization of educational work.

### Theoretical Implications:

This synthesis reaffirms the relevance of socio-cultural theories such as Wenger’s4 (1998) Communities of Practice and Stubbing et al.39 (2018) application of the figured worlds framework in medical education, both of which highlight identity as relational, dynamic, and constructed through engagement with communities and cultural practices. The former helps explain how identities are co-constructed through mentorship, dialogue, and peer networks. Many educators described informal spaces, like faculty learning groups or peer collaborations, as critical in affirming their emerging roles.12 Fong and Jones27 (2024) provide further support for this perspective, demonstrating how longitudinal mentorship and engagement within peer networks enabled novice clinician-educators to renegotiate their initial clinician-centered self-concepts and progressively internalize the role of an educator.

From a theoretical perspective, the study also extends the conversation to Bloom’s affective domain, where identity formation is shaped by appreciation, emotional reward, and belonging. Such insights align with contemporary scholarship as outlined by Rodgers et al.40 that frames educator identity as not only cognitive and social but also deeply emotional, which was publicized by Kelchtermans et al.41 The presence of impostor feelings, identity dissonance, and marginalization calls for faculty development models that address both skill acquisition and identity support.13,35 Bartle et al.26 in 2025, strengthened this argument by illustrating how structured faculty development programs that embed reflective practice and foster community belonging create conditions where identity work is simultaneously cognitive, social, and affective, thus reinforcing socio-cultural and emotional dimensions of PIF.

### Comparison with Teacher Identity in Other Contexts:

While educator identity has been extensively studied in undergraduate and school-based teaching, health professions educators experience distinctive identity challenges. Most notably, many enter academia after significant clinical training and experience, often with limited pedagogical exposure as demonstrated by Riveros-Perez & Rodriguez-Diaz29 in 2017. This contrasts with schoolteachers or university lecturers who often enter teaching as a primary career identity.

The tension between disciplinary/clinical identity (e.g., physician, nurse) and educational identity is particularly acute in health professions. These professionals often face conflicting expectations, balancing service, research, and teaching as shown by Browne et al.30 and struggle to position teaching as an equal component of their professional self, as illustrated by Sethi and colleagues.28 In contrast, undergraduate health professions educators typically operate in environments that are more pedagogically oriented, even if similarly under-resourced, as shown by Hays et al.42 They imply that while health professions education is embedded within the healthcare system, it applies educational principles in environments that may not be primarily designed for teaching.

Interestingly, literature from both medical education33 and higher education42 more broadly highlights the importance of mentorship, emotional validation, and reflective practice in professional identity formation. Within health professions education specifically, however, the path to legitimacy for educators may often become longer and more conflicted due to the prevailing complexity and dominance of clinical cultures, which tend to undervalue teaching in favour of research or clinical expertise. Fong and Jones27 (2024) highlight this challenge by showing that novice clinician-educators initially construct their professional identities almost exclusively through their clinical background, requiring sustained mentorship to develop legitimacy as educators. Similarly, Bartle et al.26 in 2025 demonstrated that structured faculty development can mitigate these tensions by embedding reflective practices and community connections, thereby accelerating the recognition of teaching as a core professional identity alongside clinical and research responsibilities.

Local work on digital professional behaviour underscores the relevance of contextual influences on identity trajectories within Pakistan43

### Limitations

This systematic review is subject to a few limitations. First, while the search strategy was comprehensive, it was limited to peer-reviewed, English-language publications, which may have excluded relevant research published in other languages or gray literature. Additionally, most of the included studies were conducted in Western academic contexts, potentially limiting the transferability of findings to regions with differing educational cultures, structures, and faculty development systems.

Another limitation is the inherent heterogeneity in study designs and participant groups across the included literature. While qualitative methodologies provide rich insight into identity formation, the variability in theoretical frameworks and depth of analysis makes comparative synthesis challenging. Some studies offered only peripheral attention to identity processes, even when included based on high relevance to the research questions.

Furthermore, the review focused exclusively on self-reported experiences. The absence of longitudinal or ethnographic studies limits the understanding of how identities evolve over time in response to changing roles, institutional expectations, or professional disruptions such as burnout or career transitions.

### Implications

The findings of this review hold several practical implications for faculty development in health professions education. Firstly, identity formation must be recognized as a core component of faculty development, not merely an implicit outcome. Programs should incorporate well-regulated and structured training through reflective exercises, narrative practices, and mentorship structures to help educators articulate and reconcile their professional identities. Recent work underscores the value of longitudinal mentorship in supporting novice clinician-educators to navigate the tensions between clinical and educator identities, highlighting mentorship as an essential enabler of identity transformation.27

Institutional support plays a critical role in legitimizing teaching identity. This includes aligning career advancement structures with educational excellence, publicly recognizing teaching contributions, and embedding educator roles into strategic planning process.

Embedding frameworks such as the Academy of Medical Educators’ Professional Standards*44* within faculty development and appraisal systems, clarifies educator competencies across teaching, scholarship, and leadership, thereby strengthening professional identity and legitimacy.

Leadership training and peer-led learning communities can further foster shared meaning-making and a sense of belonging among educators. Faculty development programs that integrate structured reflection, opportunities for application, and sustained community engagement have been shown to consolidate educators’ identity, demonstrating the importance of intentional program design.26

## CONCLUSION

This review highlights that professional identity formation in health professions educators is a complex, multi-dimensional process shaped by individual, institutional, and societal factors. Transitioning from a clinician or a researcher to an educator requires more than acquiring teaching skills—it demands legitimacy, reflection, and community. Evidence shows that novice clinician-educators often require sustained mentorship to renegotiate their primarily clinical self-concept and progressively internalize the role of an educator as outlined by Fong & Jones27 in 2024. Likewise, faculty development programs that incorporate reflective practice, opportunities for application, and sustained peer engagement have been shown to accelerate identity consolidation and legitimize educational roles within institutional cultures, as shown by Bartle et al.26 By understanding the enablers and barriers to identity formation, institutions can better support faculty through intentional design of development programs, recognition systems, and relational learning spaces. Ultimately, strengthening educator identity is vital for cultivating a sustainable and impactful academic workforce in health professions education.

### Recommendations

Future research should prioritize longitudinal studies that trace identity development over time and across career stages. Additionally, there is a need to explore how socio-cultural and institutional contexts influence identity formation in underrepresented regions or resource-constrained settings. Integrating emotional and relational frameworks into identity research may also yield deeper insights into educator wellbeing and resilience.
